# Investigation of the association between the Enferplex bovine tuberculosis antibody test and the future risk of bovine tuberculosis in irish cattle in infected herds: a pilot field study

**DOI:** 10.1007/s11259-023-10200-3

**Published:** 2023-08-17

**Authors:** Jamie M. Madden, Miriam Casey-Bryars, Simon J. More, Damien Barrett, Eamonn Gormley, Eoin Ryan

**Affiliations:** 1 Centre for Veterinary Epidemiology and Risk Analysis (CVERA), University College Dublin, Dublin, Ireland; 2 Ruminant Animal Health Division, Department of Agriculture, Food and the Marine, Backweston, Co. Kildare Ireland; 3 Tuberculosis Diagnostics and Immunology Research Laboratory, School of Veterinary Medicine, University College Dublin, Dublin, Ireland; 4 Animal Welfare Division, Department of Agriculture, Food and the Marine, Kildare St, Dublin 2, Ireland

**Keywords:** Bovine tuberculosis, Antibody test, Pilot field study

## Abstract

The Single Intradermal Comparative Tuberculin Test (SICTT) and the interferon-gamma (IFN-γ) assay are the approved diagnostic tests for bovine tuberculosis (bTB) in Ireland. The aim of this pilot study was to explore if there was any added diagnostic benefit from applying the Enferplex bTB test (an antibody test) in severe bTB herd breakdowns after the removal of cattle that had tested positive to the SICTT and the IFN-γ test. In addition to the normal bTB testing and management protocols, the animals in these herds that tested negative to SICTT and the IFN-γ test were followed forward for a period of two years. All animals were tested by Enferplex at enrolment. The time to subsequent bTB detection (diagnosed with SICTT/IFN-γ tests or detection of visible lesions at routine slaughter) for animals that tested positive or negative to the Enferplex bTB test at the start of the study was compared using Kaplan–Meier survival curves and Cox based survival models. Of the 484 enrolled animals (from 11 herds), 171 (35.3%) and 151 (31.1%) initially tested positive in the Enferplex assay under the high sensitivity and high specificity interpretation settings respectively. The results of the survival analysis showed that there was no difference in the survival time to a positive diagnosis with bTB during the follow-up period between animals initially classified as positive and negative by the Enferplex test. Further research is warranted to explore the potential benefit of using the Enferplex test in other scenarios.

**Supplementary Information:**

The online version contains supplementary material available at 10.1007/s11259-023-10200-3.

## Introduction

Bovine tuberculosis (bTB), principally caused by infection with *Mycobacterium bovis* (*M. bovis*), is present in many countries including Ireland (More 2019; Olea-Popelka et al. 2017). It can cause significant economic losses in livestock production and if left uncontrolled can pose a risk to public health. In Ireland, the Single Intradermal Comparative Tuberculin Test (SICTT) is the primary screening test for detection of bTB in infected herds. The interferon-gamma (IFN-γ) assay is also used, as an ancillary test, interpreted in parallel with SICTT in severe bTB breakdowns (BDs), to improve the sensitivity of detection of bTB (Clegg et al. 2019). As both tests are imperfect with respect to diagnostic sensitivity and specificity, there is a need to investigate other diagnostic bTB tests and test combinations that may offer potential benefits in terms of identifying additional infected cases. Both the SICTT and the IFN-γ test are based on the cell-mediated immune (CMI) responses.

Serological tests that detect humoral antibody responses against *M. bovis* antigens offer an alternative supplementary test for detecting bTB infection. Among those available, the Enferplex bTB antibody test (Enferplex bTB test) is a chemiluminescent multiplex serology test for detecting antibodies in bTB infected cattle based on an array of *M. bovis* antigens printed onto the surface of wells in microtitre plates (O’Brien et al. 2023). The antibody responses to individual antigens can be detected and quantified, with the dichotomous positive or negative test results used to support bTB control efforts.

The objective of this study was to explore the possible added benefit of using the Enferplex test in bTB herd BDs after the removal of cattle that had tested positive to the SICTT and IFN-y tests. That is, could the Enferplex test detect additional infected animals that were not picked up by the current standard test regime (i.e., diagnostic testing in parallel). This was investigated in a longitudinal pilot field study where animals that had tested negative to the SICTT and IFN-y in newly disclosed bTB herd BDs were enrolled and tested with Enferplex. The bTB status of each animal, as determined by the SICTT, IFN-γ or detection of visible lesions at routine slaughter, was recorded at the end of a two-year period of follow-up. The final bTB status was stratified by those that tested negative and positive to the Enferplex test at enrolment. The reason for conducting this study was to carry out an initial evaluation of the association between the results of the Enferplex test with follow-up bTB testing, in order to inform and provide an initial evidence base for any decision process on whether a larger scale field study might be worth carrying out.

.

## Methods

### Study design

In this pilot field study, 11 herds with a size of at least 80 cattle, which had newly entered a severe bTB BD were recruited into the study at the start of 2020. Herds were selected from two counties (Clare and Kerry), for operational reasons, so that only two Regional Veterinary Offices (RVOs) needed to be involved. Herds were selected in consultation with local RVO veterinary staff. In severe BDs, typically with five or more reactors, IFN-γ testing is compulsory for cattle in the exposed cohort aged over six months. It is carried out shortly after the initial disclosure of reactors in the SICTT herd test. Usually, the time from SICTT to IFN-y testing is two to four weeks, depending on logistical and herd management issues. Each infected herd was managed by routine procedures as part of the Irish bTB eradication programme and if animals were slaughtered, they routinely underwent normal post-mortem checks for lesions as part of the food safety post-mortem veterinary inspection. The SICTT and collection of blood samples on which the blood tests were carried out was conducted as part of the Irish national bTB eradication programme, which is subject to the EU trade Directive 64/432/EEC, and which governs the nature and frequency of testing. Blood sample testing was approved by the University College Dublin Animal Research Ethics Committee (AREC-E-16-34-Gormley). An extra serum tube of clotted blood was immediately collected for the Enferplex test while the vacutainer needle remained in place from the IFN-γ test. The study period for each animal commenced on the date of the IFN-γ test (‘the date of enrolment’), and the study was limited to animals that tested negative at this IFN-γ test (‘the study animals’). Further details on the Irish bTB testing programme can be found in the Supplementary Material.

Study animals were then followed-up from the enrolment date until the end of follow-up (either date of a positive bTB diagnosis or date of right-censoring (either date of death as a result of routine slaughter, for reasons unrelated to bTB diagnosis or date of end of study (17th February 2022), whichever occurred first). An animal was classified as bTB positive during follow-up if test positive to the SICTT, or a subsequent follow-up IFN-γ test or if visual lesions were detected at routine slaughter, which were laboratory confirmed as *M. bovis* infection. For this study, we utilised data from the Irish national bTB eradication programme (Animal Health Computer System (AHCS), Animal Identification and Movement System (AIM) (Tratalos et al. 2020), Laboratory Information Management System (LIMS), which included results from herd-level skin tests, animal-level laboratory tests (from animals with suspect bTB lesions) and the animal-level IFN-γ blood tests.

### Enferplex

Serum samples were tested using the Enferplex according to the manufacturer’s instructions (Enfer, Naas, Ireland). The image from the Enferplex bTB test was analysed and data reduced to determine sample status using an “Enferplex bTB Macro” which explored 11 individual antigens. These are Rv2975 synthetic peptide p652; PPDb; recombinant Rv2873; recombinant Rv2975; Bovine TB cocktail; recombinant Rv2031c-Rv1886c fusion protein; recombinant Rv3875-Rv3874 fusion protein; recombinant Rv3874-Rv3875 fusion protein; recombinant Rv2626; recombinant Rv0251c and recombinant Rv2031c (O’Brien et al. 2023). The assay has been developed using the Enferplex Multiplex Platform which allows multiple results to be generated from a single sample including a positive and negative result under different high specificity and high sensitivity settings. Samples that give positive reactions to two or more antigens are deemed to be ‘positive’, while samples that recognise zero or one antigen are deemed to be ‘negative’. The two separate positivity thresholds were established by the manufacturer by selecting antigen cut-offs appropriately to give the high sensitivity setting with a target sensitivity of 98.0% and the other, high specificity setting with a target specificity of 99.5%. In addition, a count of the number of antigens (out of the 11 tested) that were test positive was also extracted as a continuous variable (for both settings). Further details on the test and the antigens used in the Enferplex test is available elsewhere (O’Brien et al. 2023).

### Statistical analysis

All of the data are expressed as the median (interquartile range (IQR)) or as percentages where appropriate. The number and percentage of animals that subsequently tested positive either by SICTT, follow-up IFN-γ testing or culture were calculated along with a breakdown by the “high sensitivity” and “high specificity” interpretations to generate Enferplex positive or negative dichotomous results.

As we had time-to-event data, a survival analysis was undertaken. Here, the primary endpoint in the analysis was bTB status (positive or negative as described previously) of the study animal at the end of follow-up. For each animal, animal-time (in days) was calculated from the enrolment date to the end of follow-up. Unadjusted bTB detection (time to subsequent positive test) between those classified as positive and negative for the two Enferplex groups (high sensitivity and high specificity settings) was assessed using Kaplan–Meier survival curves (log-rank test). For survival estimates, deaths not relating to bTB removal were censored at the time of death (i.e., animals slaughtered for other reasons, and not slaughtered because of bTB). As these are artificial deaths, this is a reasonable approach. Those animals that reached the study end point (17th February 2022) and not classified as bTB positive, were also censored.

The Enferplex test results above (“high sensitivity” and “high specificity”) are dichotomous variables i.e., positive or negative. The hazards of dichotomising variables are well documented, such as significant loss of information and reduced power in a modelling setting (Altman and Royston 2006; Royston et al. 2006). The dichotomous Enferplex variables are constructed from continuous measures made up of the number of antigens that are recognised immunologically in infected animals. In addition to analysing the dichotomous results, we investigated the association between the number of Enferplex positive antigens and bTB detection during follow-up, using a regression model. A range of variables (age, sex, breed type, herd size and Enferplex results) and different models were explored using multivariate Cox proportional hazards models to estimate adjusted hazard ratios (HRs) and 95% confidence intervals (CIs) for associations between the covariates and bTB detection. Many regression models assume that the predictors are linearly related to the outcome which can lead to poor inference or predictions if non-linear relationships exist. Here, we explored smooth relationships for continuous variables using restricted cubic regression splines to test for linearity and to allow flexibility in its estimation. Different models were also compared using Akaike Information Criterion (AIC) values and nested models were formally tested using a likelihood ratio test (LRT). The proportional hazards assumption was explored in the final model by visually inspecting Schoenfeld residual plots. All analyses were conducted using R version 4.2.1 (R Core Team, 2022). A p-value of < 0.05 was considered as statistically significant. Survival analysis was conducted using the *survival* (Therneau 2022) and *rms* (Harrell 2022) packages.

## Results

A total of 626 cattle from the 11 herds were tested with the Enferplex test. After removal of 142 animals that were positive to the initial IFN-γ test or, positive to the SICTT on or prior to the enrolment date, the animals identified for follow-up comprised of 484 cattle that were negative to an initial IFN-γ test between 24th February 2020 and 20th April 2020. The characteristics of these animals and herds are presented in Table 1. The majority of animals were female (96.7%), dairy cows (74.8%) with a median age of 4.4 (IQR: 3.0 to 7.1) years. They predominantly originated from dairy herds (54.5%) with a median of 28 (IQR: 14 to 60) bTB cases in the BD. The median herd size (based on the number of animals in the herd during tests) was 162 (IQR: 108 to 202) and median BD duration was 357 (IQR: 192 to 486) days for all herds. The number of cattle sampled for testing for IFN-y, and thus Enferplex tested, was less than the total herd size of all 11 herds, as the guidelines on the use of IFN-y in the Irish bTB eradication programme is generally restricted to cattle aged over six months and those in the bTB exposed cohort of cattle. For example, if a herd had a non-exposed group of cattle on an out-farm, these are not usually IFN-y tested.

**Table 1:** Characteristics of the 484 animals in the 11 herds

Variable		n (%)/median (IQR)
**Animal level data**		
Age	Median (IQR)	4.4 (3.0 to 7.1)
Sex	Female	468 (96.7)
	Male	16 (3.3)
Breed type	Beef	122 (25.2)
	Dairy	362 (74.8)
No. of SICTT during study	Median (IQR)	5 (3–5)
**Herd level data**		
Total SICTT reactors	Median (IQR)	23 (9 to 45)
Total SICTT reactors with lesions	Median (IQR)	5 (2 to 8)
Total IFN-γ cases	Median (IQR)	5 (4 to 14)
Total/All cases (SICTT + slaughter + IFN-γ)	Median (IQR)	28 (14 to 60)
Total animals over all tests	Median (IQR)	162 (108 to 202)
Total SICTT during BD	Median (IQR)	1028 (444 to 1244)
Breakdown duration (days)	Median (IQR)	357 (192 to 486)
Herd type	Dairy	6 (54.5)
	Other	1 (9.1)
	Suckler	4 (36.4)

SICTT: Single Intradermal Comparative Tuberculin Test; IFN-γ: interferon-gamma

Of the enrolled 484 animals that were followed-up, 171 (35.3%) and 151 (31.1%) tested positive to the Enferplex at enrolment under the high sensitivity and high specificity setting respectively. A descriptive breakdown of these animals by follow-up testing results is presented in Table 2. Overall, there were 57 (11.8%) animals diagnosed with bTB during follow-up with the majority detected with the SICTT (61.4%). Two of these reactor animals had visible lesions disclosed at post-mortem examination. Among the cattle slaughtered routinely, a single animal was observed to have suspect bTB lesions which was subsequently confirmed as bTB. Table 2 shows that there was no difference between the high sensitivity and high specificity settings when comparing the bTB case disclosure method, i.e., culture or visible lesion rate at post-mortem for reactors. There was a minimal (n = 20) difference in the bTB outcome when comparing animals positive between the Enferplex test using the high sensitivity and high specificity settings.

**Table 2:** A breakdown of test results during follow-up testing for bTB for all 484 study animals stratified by Enferplex high sensitivity/high specificity result (percentage of animals are included row-wise but the total column percentages are column-wise)

		High sensitivity		High specificity		
Variable		Negative n (%)	Positive n (%)	Negative n (%)	Positive n (%)	Total n (%)
bTB case disclosure method (n = 57)	IFN-γ	13 (61.9)	8 (38.1)	13 (61.9)	8 (38.1)	21 (36.8)
	Lesions detected at routine slaughter confirmed as TB	1 (100.0)	0 (0.0)	1 (100.0)	0 (0.0)	1 (1.8)
	SICTT	23 (65.7)	12 (34.3)	23 (65.7)	12 (34.3)	35 (61.4)
Culture (n = 60)	Negative	37 (64.9)	20 (35.1)	37 (64.9)	20 (35.1)	57 (95.0)
	Positive	1 (33.3)	2 (66.7)	1 (33.3)	2 (66.7)	3 (5.0)
Visible lesions post-mortem (reactors) (n = 57)	Negative	36 (65.5)	19 (34.5)	36 (65.5)	19 (34.5)	55 (96.5)
	Positive	1 (50.0)	1 (50.0)	1 (50.0)	1 (50.0)	2 (3.5)
Final bTB status at end of follow up (n = 484)	Negative*	276 (64.6)	151 (35.4)	296 (69.3)	131 (30.7)	427 (88.2)
	Positive	37 (64.9)	20 (35.1)	37 (64.9)	20 (35.1)	57 (11.8)

Culture: if a lesion was sent for culture (from any of the bTB case disclosure methods). Visible lesions from animals already deemed reactors before entering slaughterhouse (these are either IFN-γ or SICTT positive animals). *Only difference of n = 20 animals became positive when high sensitivity setting was applied (compared to high specificity). SICTT: Single Intradermal Comparative Tuberculin Test; IFN-γ: interferon-gamma

**Fig. 1:**
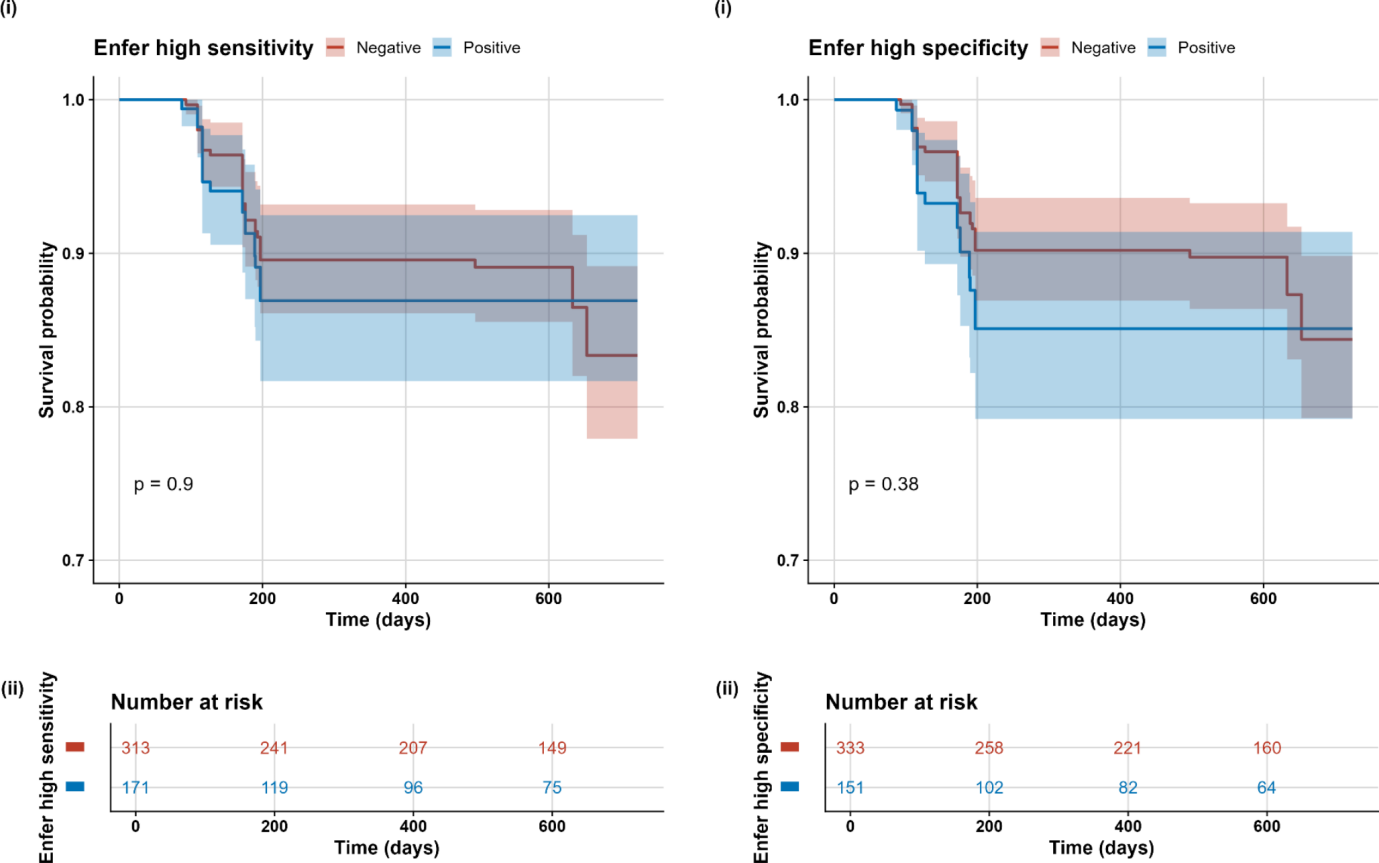
(i) Kaplan–Meier estimated bTB survivor functions for cattle that were classified as either positive and negative to the Enferplex test under high sensitivity (left) and high specificity (right) settings. The shaded regions represent 95% confidence bands. (ii) The number at risk table associated with the Kaplan-Meier curve represents the number of animals remaining in the study (and “at risk”) at selected timepoints that have not yet been detected with bTB. The figures decrease over time as animals (a) test positive for bTB and are mandatorily removed and (b) are removed due to censoring (e.g., animals sent to slaughter without bTB or study end date)

Kaplan–Meier estimated survivor functions plots were used to compare those animals classified as positive and negative by the Enferplex high sensitivity and high specificity settings (Fig. 1). Both plots show that there was no measured difference in the risk of a positive bTB diagnosis during follow-up when comparing those animals classified as positive and negative by the Enferplex. Separately, the number of Enferplex positive antigens were examined and boxplots of these continuous measures were plotted with the raw points jittered and colour coded by the last recorded bTB status of the cattle at the end of follow-up (see Supplementary Material). There was no apparent difference in the number of Enferplex antigens that were recognized by sera from positive and negative outcome cattle. To explore this further, a selection of multivariable Cox based survival models were applied to investigate the association between the number of Enferplex positive antigens recognised and bTB detection during follow-up (see Supplementary Material). The best fitting model (as determined by AIC and LRT) included age (non-linear form), sex, breed type (beef or dairy) and herd size. A model that included the Enferplex results (either dichotomous or continuous variations) did not improve model fit. For brevity, the output from a model including a Enferplex result is included in the Supplementary Material. It shows that there was no association between the number of positive antigens and future risk of bTB (HR: 0.99 (95% CI: 0.88–1.13) while age, males, dairy cattle and herd size were statistically significantly associated with bTB.

## Discussion

In this pilot field study, we applied the Enferplex test to cattle that had tested negative to the SICTT and the IFN-γ test from newly disclosed bTB infected herds. During the period of follow-up, we compared the time to bTB detection among cattle that tested positive and negative to the Enferplex bTB test at time of enrolment. We found that the future risk of bTB (as classified by the current suite of tests normally used in the Irish bTB eradication programme) was similar for animals that tested negative or positive to the Enferplex under both high sensitivity and high specificity settings. Additionally, in Cox based survival models, the Enferplex results were not statistically significantly associated with future risk of testing positive for bTB.

Tuberculosis in cattle induces a spectrum of immunological responses where the balance of the host immune response shifts over time. Conventionally, it was understood to shift from a predominantly CMI T helper 1 (Th1) response during the early stages of infection towards a Th2 humoral antibody-based response as the disease progresses (Welsh et al. 2005). However, it has been reported that cattle antibody responses to the immunodominant antigen MPB83 can be detected early post-infection (4 weeks) and appear to be influenced by route of infection in experimentally infected cattle (Waters et al. 2006). The SICTT and the IFN-γ diagnostic tests target the CMI response to *M. bovis* whereas the Enferplex test measures the Th2 antibody response. In large scale field studies, it has been shown that there is considerable overlap with animals testing positive with CMI and antibody tests (Clegg et al. 2011). However, we cannot assume that *M. bovis* infected animals with a positive response in antibody tests will always test SICTT or IFN-γ positive. This highlights a challenge in determining the role for antibody tests within a well-managed disease control programme that targets and removes reactor animals based on positive CMI responses (McCallan et al. 2021). Classifying the true *M. bovis* infection status of cattle in our study would ideally require a perfect diagnostic test; in its absence, infection status was inferred using existing tests, accepting their known limitations.

A recent study on the diagnostic accuracy of the Enferplex test, co-authored by the manufacturers, found that the overall sensitivity and specificity of the test was 93.9% (95% CI: 89.9–96.4) and 99.7% (95% CI: 99.5–99.8) respectively (O’Brien et al. 2023). In that study, the testing was carried out using two well-defined reference populations; positive and negative reference sera collected from animals with known *M. bovis* infection or from animals in herds free of bTB respectively. Although the samples collected from animals in the current study were from infected herds with at least five SICTT reactors, it is reasonable to assume that many of these animals did not have a similar risk-profile as animals in the earlier study. A similar study from Belgium, which explored the Enferplex bTB test in 308 animals, found that its performance was 58.7% (95% CI: 51.4–66.1) and 95.1% (95% CI: 92.1–97.3) for the sensitivity and specificity respectively (under high sensitivity setting), relative to SICTT, which the authors highlighted was lower than those established by the manufacturer (Moens et al. 2023). A further study, (McCallan et al. 2021), based on a cohort of cattle (n = 922) from Northern Ireland bTB reactor herds eligible for inclusion in the IFN-γ testing scheme (higher risk herds), used plasma samples taken before tuberculin injection to investigate serology as a potential replacement for SICTT. This was not directly comparable with our work or (Moens et al. 2023; O’Brien et al. 2023), as bespoke two and four antigen Enfer assays on plasma not boosted by tuberculin were used. However, resonating with our findings, very few of the cattle which subsequently tested positive on SICTT or IFN-γ with subsequent confirmation of *M. bovis* infection from lesions collected at slaughter, were detected by the bespoke Enfer assay. This current study has a different design to (McCallan et al. 2021; Moens et al. 2023; O’Brien et al. 2023), as we aimed to understand whether the Enferplex could be used to detect infected cattle which were missed by the official tests. Our study population comprised cattle, within herds undergoing bTB BDs with at least five SICTT reactors, which tested negative in SICTT and IFN-γ tests. Rather than comparing contemporaneous Enferplex and SICTT, we assessed whether the Enferplex result predicted a future diagnosis of bTB with the official tests or detection of a lesion at routine examination at slaughter.

The context in which the serology testing was carried out is important when interpreting our findings. We cannot generalise the results beyond the current study design setting. The field setting was a particularly challenging test of the utility of Enferplex for three reasons. First, cattle testing positive to SICTT and the IFN-γ test were already removed. Therefore, we were investigating whether the Enferplex could detect infected animals that were missed as false negatives by the authorised tests. Secondly, due to severity of the bTB breakdowns, our study herds were subjected to intensive tuberculin testing (repeated presentation of tuberculin antigens to the immune system). As a result, we cannot rule out that some animals may have developed an antibody response to a range of antigenic components of tuberculin rather than being truly infected with bTB, which may have resulted in a positive test result on the Enferplex test. Finally, as we were examining the association between a test result at study enrolment with an outcome at the end of follow-up, a limitation of this study is temporal bias, in that we don’t know if those animals that were detected as bTB positive during follow-up were infected at the time of their Enferplex test or subsequently during follow-up. Further research is warranted to investigate the potential benefits of using the Enferplex test in other diagnostic settings. This would include modelling the impact of different combinations of bTB tests in various epidemiological scenarios. Given the logistical challenges of carrying out large scale field evaluations of bTB tests, such an approach could support policy makers in exploring how best to optimise diagnostic testing protocols.

### Electronic supplementary material

Below is the link to the electronic supplementary material.

Supplementary Material 1

## Data Availability

The datasets analysed during this study are available from the Department of Agriculture, Food and the Marine, but are subject to data protection regulations and limitations (https://www.gov.ie/en/organisation-information/ef9f6-data-protection/).
