# Supplementary material for: Investigation of the association between the Enferplex bovine tuberculosis antibody test and the future risk of bovine tuberculosis in irish cattle in infected herds: a pilot field study
Source: Vet Res Commun. 2023 Aug 17;48(1):555–61. doi: 10.1007/s11259-023-10200-3 (PMC10811095; doi:10.1007/s11259-023-10200-3)
Supplement: Supplementary file 1 — Supplementary Material 1 [file 11259_2023_10200_MOESM1_ESM.docx]

**Supplementary Material Irish bTB programme**

In Ireland, a SICTT herd level test is applied annually to all herds. In addition to the SICTT, if animals are sent to slaughter, they undergo normal post-mortem checks for lesions as part of the food safety post-mortem veterinary inspection. If one or more positive bTB cases are detected (either by the SICTT or by the appearance of visible lesions at slaughter which are then confirmed as *m.bovis*), the herd enters a BD until it has two consecutive clear herd tests each 60 days apart. In serious BDs, typically with five or more reactors, IFN-γ testing is compulsory for cattle in the exposed cohort aged over six months. It is carried out shortly after the initial disclosure of reactors in the SICTT herd test. Usually, the time from SICTT to IFN-y testing is two to four weeks, depending on logistical and herd management issues. Definitions for standard and severe reactors can be found here [1], while more information on the IFN-γ test can be found here [2].

[1] Byrne AW, Barrett D, Breslin P, Ryan E. Can more information be extracted from bovine TB skin test outcomes to inform animal risk management? A retrospective observational animal-level study. Preventive veterinary medicine. 2022;208:105761.

[2] Clegg TA, Doyle M, Ryan E, More SJ, Gormley E. Characteristics of Mycobacterium bovis infected herds tested with the interferon-gamma assay. Preventive veterinary medicine. 2019;168:52-9.


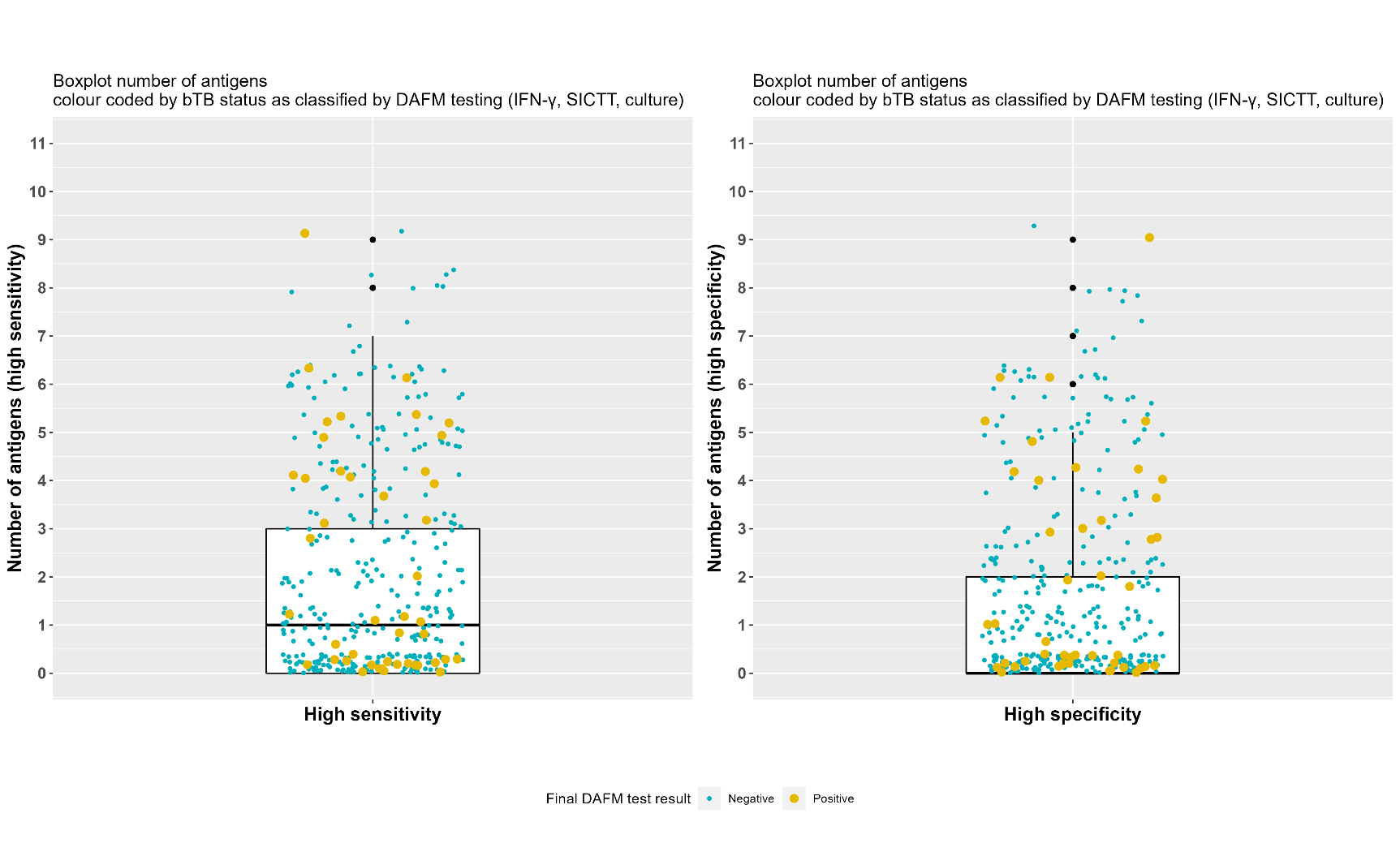


**Supplementary Material Figure 1**: Boxplots of the number of antigens from the Enferplex test under both the high specificity and high sensitivity setting. The raw data points are jittered over the boxplots and colour coded by the last recorded bTB status of the cattle at the end of follow-up (DAFM: Department of Department of Agriculture, Food and the Marine; IFN-γ: interferon-gamma; SICTT: Single Intradermal Comparative Tuberculin Test).

**Supplementary Material Modelling**

Many regression models assume that the predictors are linearly related to the outcome which can lead to poor inference or predictions if non-linear relationships exist, as they often do. Here, we explored smooth relationships for continuous variables using restricted cubic regression splines to test for linearity and to allow flexibility in its estimation. Different models were also compared using Akaike Information Criterion (AIC) values and nested models were formally tested using a likelihood ratio test (LRT). A selection of models is presented below. The best fitting model (lowest AIC) included age (non-linear form), sex, breed type (beef or dairy) and herd size. A model including Enferplex results (either dichotomous or continuous) did not improve model fit.

Table 1: A comparison of Cox models including ones with and without Enferplex results (both dichotomous and continuous (no. of antigens) were explored)

| Model | Variables | AIC | observations |
| --- | --- | --- | --- |
| Model m1 | Age, Sex | 666.2833 | 484 |
| Model m2 | Spline term 1 Age, Spline term 2 Age, Sex | 657.1191 | 484 |
| Model m3 | Spline term 1 Age, Spline term 2 Age, Sex, Breed type, Herd size | 626.7340 | 484 |
| Model m4 | Spline term 1 Age, Spline term 2 Age, Breed type, Herd size | 631.6640 | 484 |
| Model m5 | Spline term 1 Age, Spline term 2 Age, Sex, Breed type, Herd size, High specificity result | 628.6504 | 484 |
| Model m6 | Spline term 1 Age, Spline term 2 Age, Sex, Breed type, Herd size, High specificity no. of antigens | 628.7331 | 484 |

An example of a model (Model m5 above) including an Enferplex variable which suggested there was no association between it, and bTB detected during follow-up is presented in Figure 2 below.


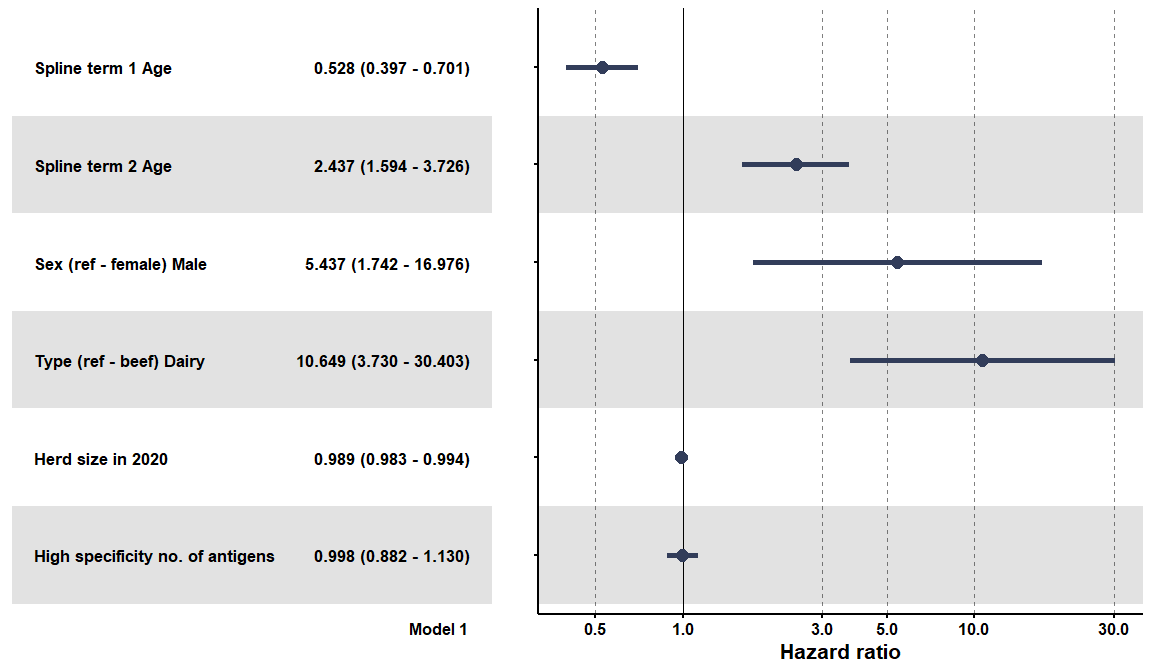


**Supplementary Material Figure 2**: Hazard ratios and interval plot from Cox based survival model for the association between predictors and bTB.


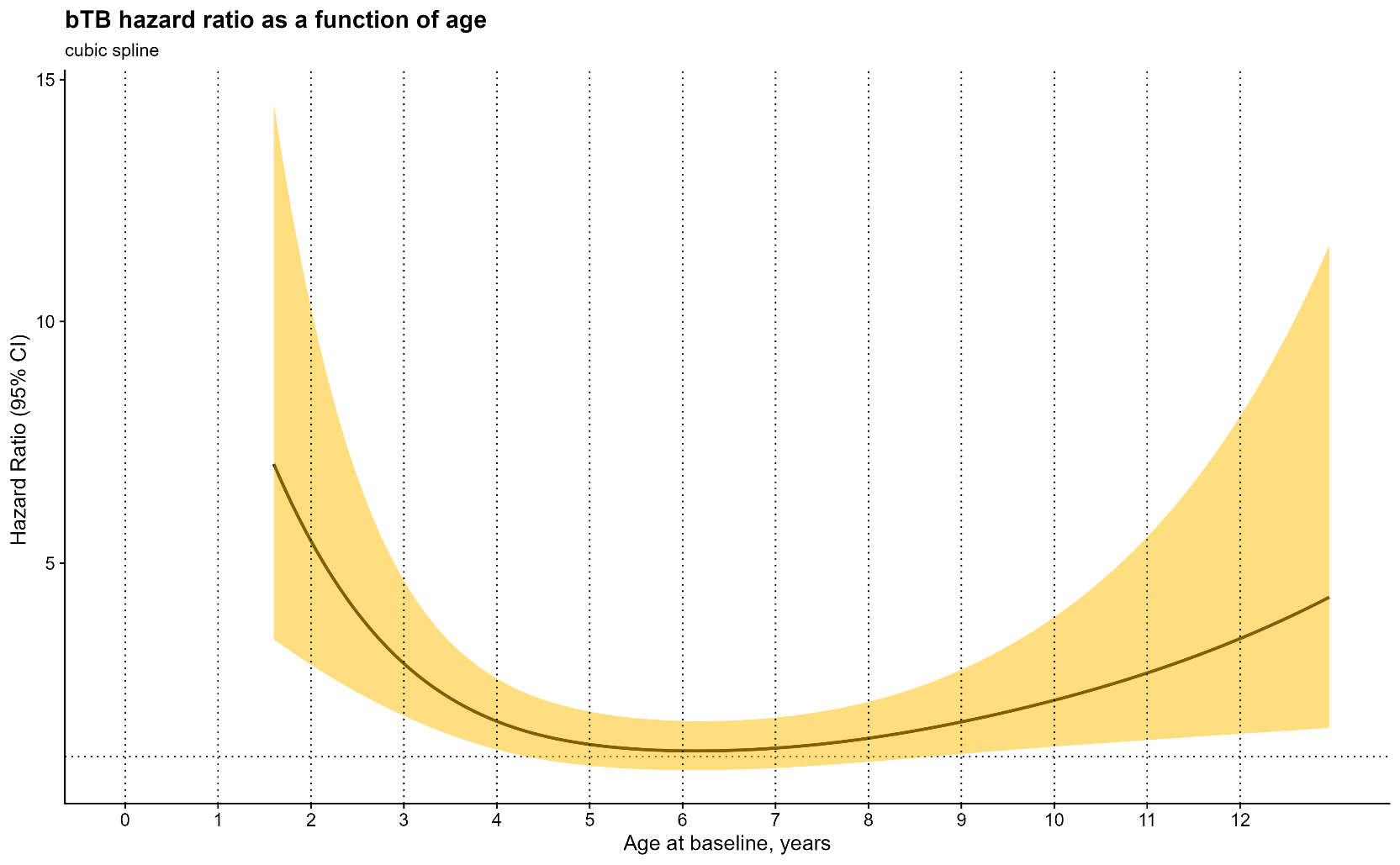


**Supplementary Material Figure 3:** Association of age and bTB using restricted cubic splines after adjustment (based on Model m5). Hazard ratios and 95% confidence bands are presented.
